# Materialistic Psychology

**Published:** 1877-08-20

**Authors:** A. Donald

**Affiliations:** Opelousas, La.


### MATERIALISTIC PSYCHOLOGY.

BY A. DONALD, M. D., OPELOUSAS, LA.

OPELOUSAS, LA., July 7th, 1877.

On page one hundred and sixty-three, (June number 1877,) I notice an article from "the London *Medical Journal*" in which Dr. Winn sums up his arguments upon the above subject. It appears to me, that the time has fully come, when there should be no want of agreement among men of science, upon the subject of mental philosophy. If we examine the *Minute Anatomy* of the human brain, and compare it with the impressions made upon the mind, we shall look in vain to find these impressions made upon brain substance. What then shall we look for, simply to find how ideas are impressed upon the mind? If we can show how this is done, it will assist us in making out the phenomena of insanity. If we assume the existence of an entity called mind or soul, (and this can be done as readily as the existence of brain) we prove it by certain attributes, which belong to no other substance in being, some of these are consciousness, perception, reflection, and reason; the brain then being the temporary residence of the soul, is constructed in relation to the phenomena, which the soul or mind has to manifest, hence if we trace out the anatomical relation between such structure and the mind, we shall find, centripetal and centrifugal nerves, the centripetal being fully charged with electricity, and, in a normal condition, transmit ideas to the mind, and the centrifugal nerves convey these impressions outwardly, it would be the greatest absurdity in the world, to suppose that the millions of impressions upon the human mind, could be photographed upon brain substance, the microscope will never demonstrate any such absurdity, the brain as such, has not the attribute of memory, perception, reflection and reason. Insanity, then, is referable to an altered condition of the centripetal and centrifugal nerves, a pathological condition—let post mortem examinations look in this direction, and these views will be sustained. Insanity comes from morbid conditions of these nerves and other brain substance, and not of a diseased mind or soul, which cannot be diseased. The attributes of mind forbid the idea of disease. If the mind could be a subject of disease, it would also be a subject of decay, and death, but, being *primal substance*, *it is uncreated* and eternal. There is no chemical reagent which can decompose it. Its organization and intellection, result from a physical construction by the allwise Architect of the universe. If the above theory of intellection and insanity is not the correct one, let anatomists and physiologists *prove the contrary*.

